# Impact of early antiretroviral treatment on sexual behaviour: a randomised comparison

**DOI:** 10.1097/QAD.0000000000002359

**Published:** 2019-09-02

**Authors:** Fiona C. Lampe, Alison J. Rodger, William Burman, Andrew Grulich, Gerald Friedland, Wafaa El Sadr, James Neaton, Giulio M. Corbelli, Sean Emery, Jean Michel Molina, Chloe Orkin, Jose Gatell, Jan Gerstoft, Kiat Ruxrungtham, Monica Barbosa de Souza, Andrew N. Phillips

**Affiliations:** a Institute for Global Health, University College London, London, UK; b Denver Public Health, University of Colorado Health Sciences Center, Denver, Colorado, USA; c The Kirby Institute, University of New South Wales, Sydney, Australia; d Departments of Medicine and Epidemiology, Yale School of Medicine, New Haven, Connecticut; e Columbia University, New York; f University of Minnesota, Minnesota, USA; g European AIDS Treatment Group, Bruxelles, Belgium; h Université de Paris, and Department of Infectious Diseases, Hopital Saint-Louis, Paris, France; i Queen Mary University of London, London, UK; j University of Barcelona and Viiv Healthcare, Barcelona, Spain; k Copenhagen University Hospital, Copenhagen, Denmark; l Chulalongkorn University, Bangkok, Thailand; m Universidade Federal do Rio de Janeiro, Rio de Janeiro, Brazil.

**Keywords:** antiretroviral therapy, condomless sex, heterosexual, HIV, MSM, transmission

## Abstract

**Background:**

Antiretroviral treatment (ART) reduces HIV infectiousness but the effect of early ART on sexual behaviour is unclear.

**Methods:**

We assessed, within the START randomized trial that enrolled HIV-positive adults with CD4^+^ cell count greater than 500 cells/μl, the effect of early (immediate) versus deferred ART on: condomless sex with HIV-serodifferent partners (CLS-D); all condomless sex (CLS); HIV transmission-risk sex (CLS-D-HIV risk, defined as CLS-D and: not on ART or started ART <6 months ago or viral load greater than 200 copies/ml or no viral load in past 6 months), during 2-year follow-up. Month-12 CLS-D (2010–2014) was the primary outcome.

**Results:**

Among 2562 MSM, there was no difference between immediate and deferred arms in CLS-D at month 12 [12.6 versus 13.1%; difference (95% CI): −0.4% (−3.1 to 2.2%), *P* = 0.75] or month 24, or in CLS. Among 2010 heterosexual men and women, CLS-D at month 12 tended to be higher in the immediate versus deferred arm [10.8 versus 8.3%; difference:2.5% (−0.1 to 5.2%), *P* = 0.062]; the difference was greater at month 24 [9.3 versus 5.6%; difference: 3.7% (1.0 to 6.4%), *P* = 0.007], at which time CLS was higher in the immediate arm (20.7 versus 15.7%, *P* = 0.013). CLS-D-HIV risk at month 12 was substantially lower in the immediate versus deferred arm for MSM [0.2 versus 11%; difference: −10.7% (−12.5 to −8.9%), *P* < 0.001] and heterosexuals [0.6% versus 7.7%; difference: −7.0% (−8.8 to −5.3%), *P* < 0.001], because of viral suppression on ART.

**Conclusion:**

A strategy of early ART had no effect on condomless sex with HIV-serodifferent partners among MSM, but resulted in modestly higher prevalence among heterosexuals. However, among MSM and heterosexuals, early ART resulted in a substantial reduction in HIV-transmission-risk sex, to a very low absolute level.

## Introduction

In 2015, results were published from the START (Strategic Timing of Antiretroviral Treatment) [1,2] and TEMPRANO [3] randomized trials, demonstrating that, for people with diagnosed HIV, a strategy of immediate antiretroviral therapy (ART) regardless of CD4^+^ cell count reduced serious morbidity and mortality compared with ART deferral. Guidelines that had previously set CD4^+^ cell count thresholds for ART initiation were changed to recommended ART initiation for all adults with HIV at any CD4^+^ cell count level [4–6]. US guidelines had already recommended such a strategy, primarily based on evidence from observational studies [7].

Prior to this conclusive evidence of the clinical benefit of early ART, results had been accumulating regarding the protective effect of ART on HIV transmission. Initially, a number of observational studies demonstrated a marked association between the viral load of an HIV-positive person and the risk of HIV transmission to an HIV-negative partner [8–12]. In 2011, unequivocal evidence came from the HPTN 052 randomized trial, which demonstrated that use of early ART for the HIV-positive partner of serodifferent couples was associated with a 96% reduction in transmissions to the HIV-negative partner [13]. Subsequently, the PARTNER [14], PARTNER2 [15] and Opposites Attract [16] prospective observational studies provided crucial information on transmission risk specifically through condomless sex (CLS), including anal CLS, among HIV serodifferent heterosexual and MSM couples. In each of these studies, there were no within-couple linked HIV transmissions during eligible follow-up in which couples reported CLS and the HIV-positive partner was virally suppressed on ART. Together, these studies have provided the necessary evidence for assurance that HIV-positive people on ART with undetectable viral load cannot transmit HIV (Undetectable = Untransmittable; Prevention Access Campaign) [17].

As knowledge regarding the protective effect of viral load suppression on HIV infectiousness has been disseminated and publicized, and in particular since the ‘Swiss Statement’ in 2008 [18], it has been debated whether such knowledge impacts on sexual behaviour and patterns of condom use among people taking ART [19–21]. Initially the concern was that if viral suppression on ART led merely to a reduction in (rather than elimination of) HIV transmission risk, any increase by the HIV-positive individual in CLS with HIV-serodifferent partners (CLS-D) could partially negate the benefit of viral suppression on ART [19,20]. Recent findings have provided reassurance on this point, demonstrating no transmission risk in this context [14–16]. However, an increase in CLS-D associated with ART use may still be concerning in the context of suboptimal ART adherence, infrequent viral load monitoring, inaccurate knowledge of personal viral load status [22] or poor knowledge of the importance of viral suppression, a key issue in early treatment [23]. Furthermore, any changes in patterns of CLS overall may have implications for transmission of other sexually transmitted infections (STIs). It is also conceivable that reductions in condom use among HIV-positive people may affect condom use among HIV-negative people with partners of unknown HIV status.

There is, to date, little compelling evidence that ART use leads to higher levels of CLS among people with HIV. Findings from some observational studies have suggested that, in some contexts or subgroups, condom use may be influenced by knowledge of viral suppression [24–29]. However, in most studies, overall, levels of CLS-D were similar or lower among people on ART compared with those not on ART (or among people with undetectable compared with detectable viral load) [28–46]. Two randomized trials have provded data on this issue [47,48]; neither supports the hypothesis that ART use leads to increased CLS-D. However, it is important to reeavualate this association as patterns of sexual behaviour may have changed with increasing awareness of the protective effect of suppressed viral load, paritcularly since the publication of HPTN 052 in 2011 [13]. Furthermore, now that the protective effect of viral suppression on HIV transmission is assured, it is necessary to consider measures additional to CLS-D, that capture sex with risk of HIV-transmission by accounting for viral suppression [45,47–49]. When considering risk of other STIs, CLS overall is the most relevant measure.

We previously reported on sexual behaviour at enrolment in the START trial [50]. We now assess, separately among MSM and heterosexual individuals, the effect of a strategy of early ART compared to ART deferral on sexual behaviour in the first 2 years of follow-up, considering: CLS-D at month 12 (the predefined primary outcome), CLS, CLS-D with risk of HIV transmission, and other measures.

## Methods

### Study population

START was an open-label multicenter randomized trial enrolling 4684 HIV-positive people who had never taken ART and who had a CD4^+^ count above 500 cells/μl, from April 2009 to December 2013. [1] Individuals were randomized to either start ART immediately (early ART) or to defer ART until occurrence of a CD4^+^ cell count below 350 cells/μl or an AIDS event. The primary endpoint was serious AIDS or non-AIDS morbidity or mortality. On 15 May 2015, the Data and Safety Monitoring Board determined that the study question had been answered and recommended that ART be offered to all participants. Rate of the primary endpoint was lower in the immediate versus deferral arm [hazard ratio (95% CI) 0.43 (0.30–0.62) *P* < 0.001] [2].

### Transmission risk behaviour study

Participants were asked to self-complete a transmission risk behaviour questionnaire at baseline, month 4, and every 12 months subsequently. Sexual activity (vaginal or anal sex) during the previous 2 months was ascertained for: men having sex with women; MSM; women having sex with men [50]. All participants who had a risk behaviour questionnaire available within the first 2 years (at 4, 12, or 24 months) were included in this analysis. All information provided after 15 May 2015 was excluded. Sexual behaviour measures (2-month recall period) were derived at baseline and at each follow-up point, including: CLS with HIV-serodifferent (negative or unknown status) partners(s) (CLS-D); CLS; HIV transmission risk sex (CLS-D-HIV-risk) defined as CLS-D with at least one of the following: not on ART; started ART less than 6 months ago; most recent viral load greater than 200 copies/ml; no viral load in last 6 months. This last measure was intended to capture sex with a risk of HIV transmission. If a person having CLS-D was on ART, having started ART at least 6 months ago, and had most recent viral load (measured within the past 6 months) 200 copies/ml or less, then risk was assumed to be zero. Additional measures included: at least two CLS partners; at least three CLS partners; at least two CLS-D partners; at least three CLS-D partners; at least two times CLS-D; at least 10 times CLS-D; insertive CLS-D with ejaculation (men only); total number of CLS-D acts (see Table 3 footnote below for details). Injection drug use transmission risk was defined as having injected drugs in the past 2 months and having shared needles or other equipment with someone who had negative/unknown HIV status. Transmission risk beliefs were beliefs related to whether a person using HIV treatment who had an undetectable viral load could pass on HIV to another person through unprotected sex. Responses of: ‘cannot’ and ‘much less likely’ were grouped together in contrast to: ‘a little less likely’, ‘just as likely’ and ‘more likely’. The prespecified primary outcome was CLS-D at month 12. Separate analyses were prespecified for MSM, and heterosexual men and women (combined). Some measures were considered only for MSM, because of low frequency among heterosexual participants.

Transmission risk behaviour forms were updated early in recruitment. Information on CLS-D and CLS-D-HIV-risk was available from original and updated forms; information on all other outcomes was available only from the updated version. Participants were included in an analysis at a specific time point if the relevant behaviour questionnaire was available; treatment of missing values is described in the footnote of Table 3.

### Statistical methods

Baseline characteristics, and ART use and viral suppression over follow-up, were summarized according to gender/sexual orientation [50]. Subsequent analyses were carried out separately for: MSM; heterosexual men and women combined. Sexual behaviour and attitude measures were summarized by time point and randomized arm. An alternative baseline measure excluded participants diagnosed with HIV for less than 3 months who may have been reporting prediagnosis sexual behaviour. Chi-squared tests were used to compare proportions between randomized arms at months 4, 12 and 24; Mann--Whitney *U* tests were used to compare number of CLS-D acts among the subgroup of participants who were diagnosed greater than 3 months ago and reported CLS-D at baseline. Logistic regression was used to obtain odds ratios for the intervention effect on CLS-D prevalence at month 12: unadjusted, adjusted for baseline factors and stratified by baseline factors. Factors considered were: gender (heterosexual analysis only); age group (<40; ≥40 years); recruitment setting [low/middle income (Africa, Asia, Central/South America); high income (Europe/Israel; North America; Oceania)]; date randomized (<2012; ≥2012); time since HIV diagnosis (<0.5 years; ≥0.5 years); education level (less than high school; high school or above). Interaction tests were used to assess whether the intervention effect differed across subgroups.

In addition, a generalized estimating equation (GEE) logistic model was used in which data from months 4, 12 and 24 were combined, with CLS-D as the dependent variable, and time point (month 4, 12, 24), randomization arm (immediate; deferred), calendar year (year of completion of risk questionnaire, as an ordinal measure) and gender (heterosexual model only) as independent variables. An autoregressive correlation structure was used to account for repeated responses from individuals. Interaction terms between randomized arm and time point were assessed.

For all comparisons, participants were kept in their original randomized group.

## Results

Of the 4684 HIV-positive people who were randomized, 112 (2.4%) were excluded from analysis as they had not completed a transmission risk questionnaire during the 2-year follow-up. Of the remaining 4572 participants, there were 2562 MSM, 788 heterosexual men, and 1222 women. Baseline characteristics are shown in Table 1.

**Table 1:** Demographic and HIV-related factors at baseline, according to gender/sexual orientation, among 4572 participants included in the analysis^a^.

		MSM (*N* = 2562)		Heterosexual men (*N* = 788)		Women (*N* = 1222)	
	*N*	*n*	%	*n*	%	*n*	%
Age group (years)							
<30	1279	877	34.2	127	16.1	275	22.5
30–39	1559	862	33.7	268	34.0	429	35.1
40–49	1192	614	24.0	251	31.9	327	26.8
≥50	542	209	8.2	142	18.0	191	15.6
Race							
White	2034	1587	61.9	262	33.2	185	15.1
Black	1381	246	9.6	360	45.7	775	63.4
Hispanic	616	464	18.1	58	7.4	94	7.7
Asian	380	170	6.6	77	9.8	133	10.9
Other	161	95	3.7	31	3.9	35	2.9
Region							
Europe/Israel	1498	1161	45.3	202	25.6	135	11.0
South and Central America	1155	832	32.5	150	19.0	173	14.2
Africa	978	25	1.0	278	35.3	675	55.2
North America	489	302	11.8	82	10.4	105	8.6
Asia	348	147	5.7	71	9.0	130	10.6
Oceania	104	95	3.7	5	0.6	4	0.3
Year randomized							
2009–2010	978	668	26.1	152	19.3	158	12.9
2011	868	593	23.1	125	15.9	150	12.3
2012	1515	762	29.7	274	34.8	479	39.2
2013	1211	539	21.0	237	30.1	435	35.6
Education							
Less than high school	1357	266	10.4	345	43.8	746	61.0
High school/equivalent	988	549	21.4	192	24.4	247	20.2
Vocational/college	1191	887	34.6	139	17.6	165	13.5
University degree	1036	860	33.6	112	14.2	64	5.2
Time since HIV diagnosis^b^							
<3 months	915	586	23.2	132	16.9	197	16.2
3–6 months	648	430	17.0	92	11.7	126	10.4
6 months to 2 years	1424	828	32.8	249	31.8	347	28.6
2–5 years	901	458	18.2	169	21.6	274	22.6
At least 5 years	632	221	8.8	141	18.0	270	22.2
BL CD4^+^ (cells/μl)							
500–599	1451	869	33.9	239	30.3	343	28.1
600–699	1426	844	32.9	231	29.3	351	28.7
≥700	1695	849	33.1	318	40.4	528	43.2
BL viral load (log copies/ml)^b^							
<3	580	190	7.4	118	15.0	272	22.3
3–3.9	1463	770	30.1	238	30.2	455	37.3
4–4.9	2045	1274	49.8	348	44.2	423	34.7
≥5	476	323	12.6	84	10.7	69	5.7

BL, baseline.

^a^One hundred and twelve of 4684 randomized participants were not included in the substudy as they did not complete the transmission risk behaviour questionnaire at any of the three follow-up time points (months 4, 12 and 24).

^b^Missing values: *n* = 52 for time since diagnosis; *n* = 8 for baseline viral load.

### Sexual behaviour at baseline

The baseline transmission risk questionnaire was completed by 4504 of 4572 (98.5%) participants (original version *N* = 547, updated version *N* = 3957). Prevalence of CLS in the past 2 months was 39.2, 23.8, and 28.1% among MSM, heterosexual men, and women, respectively; prevalence of CLS-D was 19.9, 9.6, and 14.5%, respectively. CLS-D-HIV-risk prevalence was identical to CLS-D prevalence (by definition, as no participants were on ART at baseline).

### Antiretroviral therapy use and viral suppression over time

As of 15 May 2015, of the 4572 participants, all should have attended the months 4 and 12 visits, and 3673 (80.3%) should have attended the month 24 visit. Table 2 shows numbers completing the risk behaviour questionnaire by time point and the prevalence of ART use and viral load 200 copies/ml or less, according to the randomized arm and gender/sexual orientation. In the immediate arm, the proportion of participants who were on ART remained fairly stable from month 4 to month 24, at around 96–98%. Correspondingly, the prevalence of viral load 200 copies/ml or less increased rapidly from baseline to month 4, with some further increase to month 24. In the deferred arm, the proportion on ART increased steadily over follow-up (as more individuals reached eligibility for ART initiation), mirrored by an increase in prevalence of viral suppression.

**Table 2:** Completion of transmission risk behaviour questionnaire, prevalence of antiretroviral therapy use and viral load 200 copies/ml or less, during follow-up by randomized arm and gender/sexual orientation.

	MSM (*N* = 2562)		Heterosexual men (*N* = 788)		Women (*N* = 1222)	
	IMM	DEF	IMM	DEF	IMM	DEF
Baseline						
*N* with TRB^a^	1260	1254	372	409	594	615
On ART [*n* (%)]	0	0	0	0	0	0
Viral load^b^ ≤200 copies/ml [*n* (%)]	35 (2.8)	34 (2.7)	24 (6.5)	20 (4.9)	57 (9.6)	65 (10.6)
4 months						
*N* with TRB^a^	1237	1197	360	390	588	612
On ART [*n* (%)]	1197 (96.8)	64 (5.4)	352 (97.8)	10 (2.6)	562 (95.6)	17 (2.8)
Viral load^b^ ≤200 copies/ml [*n* (%)]	1129 (91.3)	70 (5.9)	328 (91.1)	32 (8.2)	524 (89.1)	82 (13.4)
12 months						
*N* with TRB^a^	1236	1187	351	378	583	586
On ART [*n* (%)]	1200 (97.1)	205 (17.3)	342 (97.4)	37 (9.8)	563 (96.6)	53 (9.0)
Viral load^b^ less than 200 copies/ml [*n* (%)]	1169 (94.6)	198 (16.7)	334 (95.2)	48 (12.7)	536 (91.9)	96 (16.4)
24 months						
*N* with TRB^a^	1088	1058	286	297	452	447
On ART [*n* (%)]	1065 (97.9)	416 (39.3)	277 (96.9)	72 (24.2)	435 (96.2)	96 (21.5)
Viral load^b^ less than 200copies/ml [*n* (%)]	1051 (96.6)	401 (37.9)	266 (93.0)	78 (26.3)	423 (93.6)	122 (27.3)

ART, antiretroviral therapy.

^a^TRB, transmission risk behaviour questionnaire. ART status (on/off ART) determined at the date of the relevant TRB CRF.

^b^Viral load is the latest viral load up to the date of the relevant TRB CRF. Viral load missing for five MSM and three women at baseline.

### Comparison of sexual behaviour at month 12 between randomized arms

Table 3 shows sexual behaviour by randomized arm and time point for: (a) MSM and (b) heterosexual men and women.

**Table 3:** Sexual behaviour and transmission risk beliefs, at baseline and during follow-up (months 4, 12, 24) according to randomized arm, among MSM and heterosexual men and women.

a. MSM											
		Baseline		Baseline 2^b^		4 months		12 months		24 months	
Sexual behaviour (past two months)	ARM^a^	*N*	% (*n*)	*N*	% (*n*)	*N*	% (*n*)	*N*	% (*n*)	*N*	% (*n*)
At least one CLS partner	IMM	1075	39.3 (422)	785	35.8 (281)	1142	27.6 (315)	1205	29.9 (360)	1086	33.9 (368)
	DEF	1072	39.1 (419)	803	36.6 (294)	1118	29.2 (326)	1150	31.9 (367)	1055	33.6 (355)
							*P* *=* *0.41*		*P* *=* *0.28*		*P* *=* *0.91*
At least two CLS partners	IMM	1075	16.9 (182)	785	16.7 (131)	1142	11.0 (126)	1205	12.1 (146)	1086	13.6 (148)
	DEF	1072	16.2 (174)	803	15.9 (128)	1118	11.2 (125)	1150	13.0 (150)	1055	12.7 (134)
							*P* *=* *0.91*		*P* *=* *0.50*		*P* *=* *0.53*
At least three CLS partners	IMM	1075	8.6 (92)	785	8.5 (67)	1142	5.3 (60)	1205	5.7 (69)	1086	5.7 (62)
	DEF	1072	8.1 (87)	803	8.2 (66)	1118	4.6 (51)	1150	6.6 (76)	1055	6.5 (69)
							*P* *=* *0.45*		*P* *=* *0.37*		*P* *=* *0.42*
At least one CLS-D partner^c^	IMM	1260	19.7 (248)	954	14.9 (142)	1237	11.7 (145)	1236	12.6 (156)	1088	16.3 (177)
	DEF	1254	20.0 (251)	974	16.5 (161)	1197	13.0 (155)	1187	13.1 (155)	1058	14.8 (157)
							*P* *=* *0.36*		*P* *=* *0.75*		*P* *=* *0.36*
At least two CLS-D partners	IMM	1075	7.7 (83)	785	6.9 (54)	1142	4.6 (52)	1205	4.6 (55)	1086	6.2 (67)
	DEF	1072	7.6 (81)	803	6.5 (52)	1118	5.5 (62)	1150	5.7 (65)	1055	5.8 (61)
							*P* *=* *0.28*		*P* *=* *0.23*		*P* *=* *0.71*
At least three CLS-D partners	IMM	1075	3.6 (39)	785	3.4 (27)	1142	1.7 (19)	1205	1.9 (23)	1086	2.4 (26)
	DEF	1072	3.3 (35)	803	3.0 (24)	1118	2.0 (22)	1150	2.5 (29)	1055	2.9 (31)
							*P* *=* *0.59*		*P* *=* *0.31*		*P* *=* *0.43*
At least two times CLS-D	IMM	1075	13.9 (149)	785	10.7 (84)	1142	7.2 (82)	1205	8.3 (100)	1086	11.0 (120)
	DEF	1072	13.8 (148)	803	10.6 (85)	1118	8.9 (99)	1150	9.4 (108)	1055	10.0 (105)
							*P* *=* *0.14*		*P* *=* *0.35*		*P* *=* *0.41*
More than 10 times CLS-D	IMM	1075	3.9 (42)	785	2.8 (22)	1142	1.7 (19)	1205	2.4 (29)	1086	2.4 (26)
	DEF	1072	3.1 (33)	803	1.9 (15)	1118	1.4 (16)	1150	1.7 (19)	1055	2.3 (24)
							*P* *=* *0.65*		*P* *=* *0.20*		*P* *=* *0.86*
Insertive CLS-D with ejaculation	IMM	1075	8.5 (91)	785	6.5 (51)	1142	3.7 (42)	1205	4.5 (54)	1086	5.3 (58)
	DEF	1072	8.3 (89)	803	4.7 (38)	1118	3.5 (39)	1150	4.2 (48)	1055	5.0 (53)
							*P* *=* *0.81*		*P* *=* *0.71*		*P* *=* *0.74*
At least one CLS-D-HIV-risk partner^c^	IMM	1260	19.7 (248)	954	14.9 (142)	1237	11.7 (145)	1236	0.2 (3)	1088	0.6 (7)
	DEF	1254	20.0 (251)	974	16.5 (161)	1197	12.9 (155)	1187	11.0 (130)	1058	9.4 (99)
							*P* *=* *0.36*		*P<0.001*		*P<0.001*
Believe that UVL greatly reduces transmission risk	IMM	1049	37.9 (398)			1103	42.6 (470)	1181	48.1 (568)	1057	52.9 (559)
	DEF	1048	36.8 (386)			1089	37.9 (413)	1120	40.4 (452)	1027	46.8 (481)
							*P* *=* *0.025*		*P* *<* *0.001*		*P* *=* *0.006*
				*N*	Mean (SD), median	*N*	Mean (SD), median	*N*	Mean (SD), median	*N*	Mean (SD), median
Number of CLS-D^d^ acts among subset with baseline CLS-D^e^	IMM			122	9.0 (14.5), 6	128	3.3 (7.6), 0	130	4.5 (8.8), 0	119	4.4 (10.3), 0
	DEF			135	6.1 (7.6), 6	140	3.3 (5.6), 0	149	3.2 (6.6), 0	135	2.6 (5.2), 0
							*P* *=* *0.79*		*P* *=* *0.35*		*P* *=* *0.065*
							*P* *=* *0.018^*		*P* *=* *0.76^*		*P* *=* *0.76^*

b. Heterosexual men and women											
		Baseline		Baseline 2^b^		4 months		12 months		24 months	
Sexual behaviour (past two months)	ARM^a^	*N*	% (*n*)	*N*	% (*n*)	*N*	% (*n*)	*N*	% (*n*)	*N*	% (*n*)
At least one CLS partner	IMM	882	26.2 (231)	739	25.0 (185)	896	20.0 (179)	910	19.3 (176)	733	20.7 (152)
	DEF	928	26.6 (247)	756	24.9 (188)	946	21.9 (207)	936	21.3 (199)	738	15.7 (116)
							*P* *=* *0.32*		*P* *=* *0.31*		*P* *=* *0.013*
At least two CLS partners	IMM	882	2.5 (22)	739	2.0 (15)	896	1.3 (12)	910	1.0 (9)	733	1.5 (11)
	DEF	928	2.3 (21)	756	1.9 (14)	946	1.5 (14)	936	1.1 (10)	738	1.5 (11)
							*P* *=* *0.80*		*P* *=* *0.87*		*P* *=* *0.99*
At least one CLS-D partner^c^	IMM	966	13.4 (129)	817	12.1 (99)	948	8.1 (77)	934	10.8 (101)	738	9.3 (69)
	DEF	1024	11.8 (121)	845	10.1 (85)	1002	8.8 (88)	964	8.3 (80)	744	5.6 (42)
							*P* *=* *0.60*		*P* *=* *0.062*		*P* *=* *0.007*
At least two CLS-D partners	IMM	882	1.3 (12)	739	1.2 (9)	896	0.6 (5)	910	0.6 (5)	733	0.8 (6)
	DEF	928	1.6 (15)	756	1.2 (9)	946	0.6 (5)	936	0.4 (4)	738	0.7 (5)
							*P* *=* *0.83*		*P* *=* *0.75(F)*		*P* *=* *0.75*
At least two times CLS-D	IMM	882	9.8 (86)	739	8.1 (60)	896	5.9 (53)	910	7.1 (65)	733	6.8 (49)
	DEF	928	8.4 (78)	756	7.0 (53)	946	5.8 (55)	936	5.2 (49)	738	3.8 (28)
							*P* *=* *0.93*		*P* *=* *0.089*		*P* *=* *0.013*
Insertive CLS-D											
with ejaculation (men only)	IMM	333	9.3 (31)	275	6.9 (19)	335	4.5 (15)	339	5.3 (18)	286	7.0 (20)
	DEF	365	7.4 (27)	299	6.0 (18)	367	5.7 (21)	367	4.9 (18)	297	2.4 (7)
							*P* *=* *0.46*		*P* *=* *0.81*		*P* *=* *0.008*
At least one CLS-D-HIV-risk											
partner^c^	IMM	966	13.4 (129)	817	12.1 (99)	948	8.1 (77)	934	0.6 (6)	738	0.8 (6)
	DEF	1024	11.8 (121)	845	10.1 (85)	1002	8.8 (88)	964	7.7 (74)	744	4.8 (36)
							*P* *=* *0.60*		*p<0.001*		*p<0.001*
Believe that UVL greatly reduces transmission risk											
	IMM	842	31.6 (266)			876	34.4 (301)	865	36.7 (317)	702	43.5 (305)
	DEF	888	31.1 (276)			909	28.9 (263)	904	32.2 (291)	708	39.1 (277)
							*P* *=* *0.014*		*P* *=* *0.049*		*P* *=* *0.099*
				N	Mean (SD), Median	N	Mean (SD), Median	N	Mean (SD), Median	N	Mean (SD), Median
Number of CLS-D^d^ acts among subset with baseline CLS-D^e^	IMM			92	7.7 (11.2), 6	90	2.7 (6.1), 0	92	3.5 (8.7), 0	70	3.1 (11.0), 0
	DEF			77	6.4 (8.0), 6	78	4.1 (8.3), 0	78	2.6 (8.3), 0	58	1.0 (3.7), 0
							*P* *=* *0.31*		*P* *=* *0.15*		*P* *=* *0.074*
							*P* *=* *0.25^*		*P* *=* *0.52^*		*P* *=* *0.36^*

ART, antiretroviral therapy; CLS, all condomless sex; CLS-D, condomless sex with HIV negative (or unknown status) partner(s); CLS-D-HIV-risk, HIV transmission risk sex defined as CLS-D with at least one of the following: not on ART; started ART less than 6 months ago; most recent viral load more than 200 copies/ml; no viral load in last 6 months; DEF, deferred ART; IMM, immediate ART.

^a^Randomized arm.

^b^Baseline 2 estimates exclude those diagnosed less than 3 months before completing the baseline questionnaire, who may be reporting prediagnosis sexual behaviour.

^c^Denominators are larger for CLS-D and CLS-D-HIV-risk as these measures could be derived from both versions of the risk behaviour questionnaire; other measures could be derived only from the updated version. Missing values: For binary measures of CLS, CLS-D, CLS-D-HIV-risk, among those who completed the relevant transmission risk behaviour questionnaire, missing data were assumed to be the absence of the behaviour (<5% cases). For transmission risk beliefs, missing data were excluded. For number of CLS-D acts see below.

^d^Number of CLS-D acts was approximated from grouped data as follows: 2–10 times approximated as 6; 11–30 times approximated as 20; More than 30 times approximated as 40. Number of CLS-D partners was approximated from grouped data as follows: 3–5 approximated as 4; more than 5 approximated as 8. For those who had CLS-D, if number of CLS-D acts was missing or was less than the number of CLS-D partners, the value for number of CLS-D partners was used, if this was missing, number of CLS-D partners and number of CLS-D acts was set to one.

^e^Subset is those who reported CLS-D at baseline and who had been diagnosed with HIV for at least 3 months at baseline. *P* values by chi-squared tests or Fisher's exact test (F), or Mann–Whitney U tests for CLS-D acts. ^*P* value for change from baseline in number of CLS-D acts in subset. For each analysis denominators are all participants who submitted a transmission risk behaviour questionnaire at that time point.

Among MSM, prevalence of CLS-D at month 12 (the primary outcome) was similar in the immediate and deferred arms: 12.6 versus 13.1% [difference: −0.4%, 95% CI (−3.1 to 2.2%), *P* = 0.75, chi-squared test]. The prevalence of CLS, at least two CLS-D partners, at least three CLS-D partners, at least two times CLS-D, more than 10 times CLS-D and insertive CLS-D with ejaculation at month 12 did not differ between the arms, nor did number of CLS-D acts among the subgroup diagnosed more than 3 months ago who reported CLS-D at baseline. The prevalence of CLS-D-HIV-risk at month 12 among MSM was much lower in the immediate versus deferred arms: 0.2 versus 11.0% [difference: −10.7%, 95% CI (−12.5 to −8.9%), *P* > 0.001], because of the far higher prevalence of viral load suppression on ART in the immediate arm.

Among heterosexual men and women there was some evidence that CLS-D at month 12 was higher in the immediate versus deferred arm: 10.8 versus 8.3% [difference: 2.5% 95% CI (−0.1 to 5.2%), *P* = 0.062, chi-squared test]. A similar pattern was apparent for at least two times CLS-D: 7.1 versus 5.2%, [difference: 1.9%, 95% CI (0.3 to 4.1%), *P* = 0.089, chi-squared test]. Prevalence of CLS, at least two CLS partners, at least two CLS-D partners, and insertive CLS-D with ejaculation did not differ by arm at month 12, nor did number of CLS-D acts among those diagnosed more than 3 months ago who reported CLS-D at baseline. Although prevalence of CLS-D was somewhat higher in the immediate versus deferred arm among heterosexual individuals, the prevalence of CLS-D-HIV-risk at month 12 was much lower in the immediate arm: 0.6 versus 7.7% [difference: −7.0%, 95% CI (−8.8 to −5.3%), *P* < 0.001, chi-squared test], because of viral suppression on ART.

### Comparison of randomized arms according to baseline factors: condomless sex with HIV-serodifferent partners at month 12

**Fig. 1:**
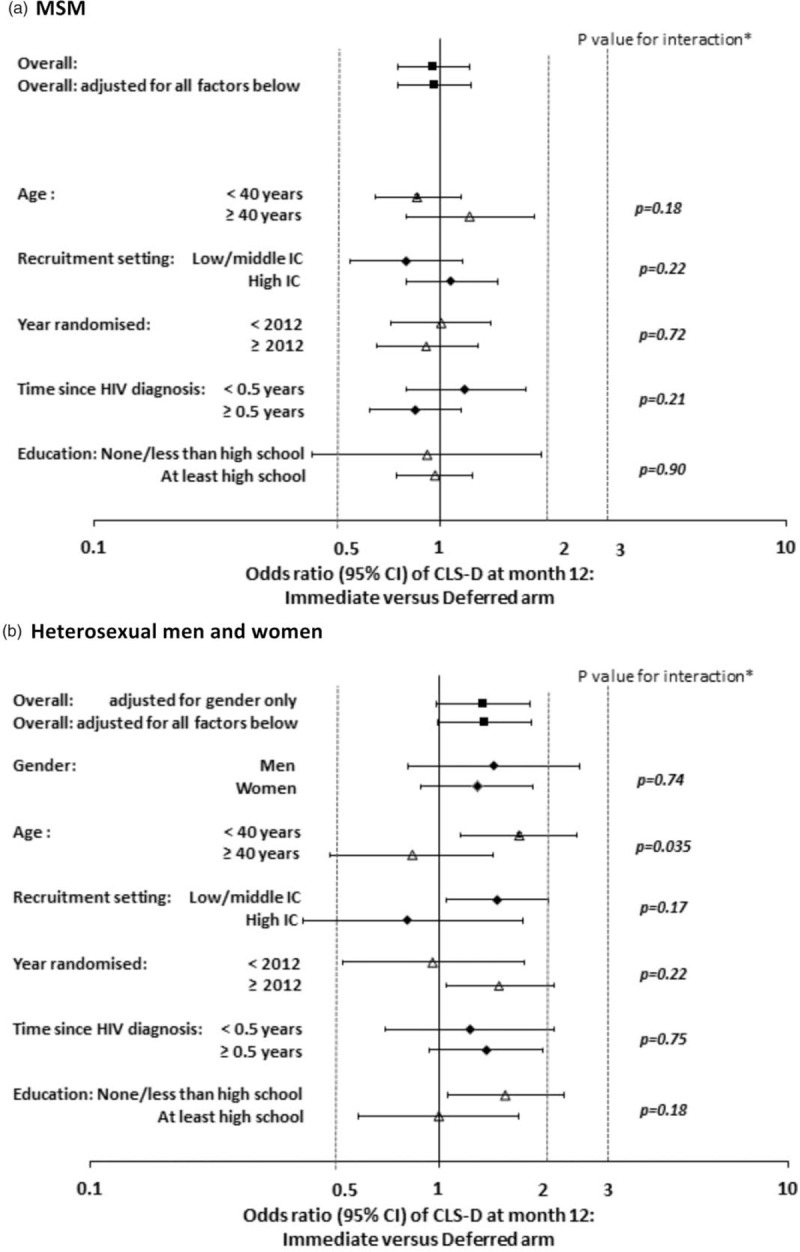
Effect of randomised group on CLS-D at month 12, according to baseline factors among MSM and heterosexual men and women.

The odds ratio (95% CI) of CLS-D at month 12 for the immediate versus deferred strategy was 0.96 (0.76 to 1.22) for MSM and 1.33 (0.98 to 1.81) for heterosexual men and women (the latter adjusted for gender; Fig. 1). Among MSM, there was no evidence that the intervention effect on CLS-D varied across subgroups. Among heterosexual men and women, the intervention effect (higher CLS-D at month 12 in the immediate versus deferred arm) was greater among participants aged less than 40 years compared to those aged 40 years or more (*P* = 0.035 for interaction).

CLS-D, condomless sex with HIV serodifferent (negative or unknown) status partner. Odds ratio from logistic regression model. ^∗^*P* value from test of interaction between each baseline factor and randomized group. Low-income/middle-income (Africa; Asia; Central/South America); high income (Europe/Israel; North America; Oceania). (a) MSM and (b) heterosexual men and women.

### Time since randomization and sexual behaviour

**Fig. 2:**
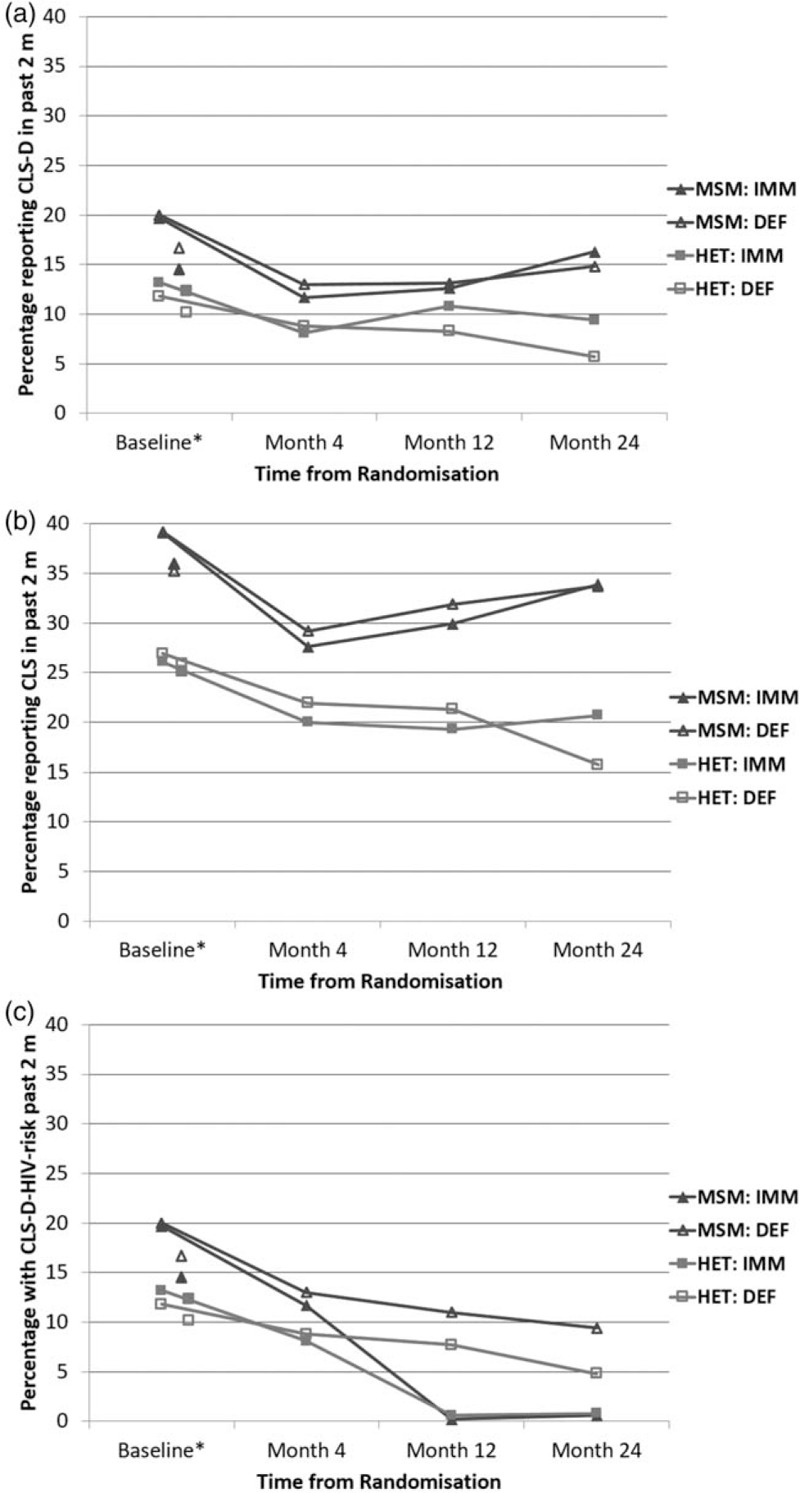
Prevalence of sexual behaviour in the past 3 months by time point and randomized arm among MSM, and heterosexual men and women: (a) CLS-D; (b) CLS; (c) CLS-D-HIV-risk.

^∗^Baseline estimates are also shown excluding participants who had been diagnosed with HIV for less than 3 months. For denominators by time point and *P* values comparing randomized arms, see Table 3. CLS-D, condomless sex with HIV serodifferent (negative or unknown) status partner; CLS, condomless sex; CLS-D-HIV-risk, HIV-transmission risk sex; DEF, deferred arm; HET, heterosexual men and women; IMM, immediate arm.

Table 3 and Fig. 2 show the prevalence of sexual behaviour measures by time point and randomized arm. The additional baseline estimates exclude participants diagnosed for less than 3 months.

Prevalence of CLS-D fell from revised baseline to month 4 in both arms, among MSM and heterosexuals. By month 24, prevalence had increased back towards the revised baseline level for MSM in both arms, and for heterosexuals in the immediate arm. For heterosexuals in the deferred arm, prevalence of CLS-D continued to fall throughout follow-up, resulting in higher CLS-D prevalence in the immediate versus deferred arm at month 24 [9.3 versus 5.6%, difference:3.7%, 95% CI (1.0 to 6,4%), *P* = 0.007, chi–squared test].

Patterns in CLS-D prevalence during follow-up were assessed formally in two multivariable logistic GEE models using data from the 4, 12 and 24-month time points. Among MSM (*N* = 7003 observations), there was no interaction between randomized arm and study time point (*P* = 0.24). In a model without an interaction term, CLS-D did not differ by randomised arm [aOR immediate versus deferred: 1.00 (0.84 to 1.19) *P* = 0.99] or calendar year [aOR per later year: 0.99 (0.91 to 1.07) *P* = 0.76] but differed by study time point: compared with month 4, odds of CLS-D was similar at month 12 [adjusted odds ratio (aOR) (95% CI): 1.05 (0,91 to 1.21)] but higher at month 24 [aOR: 1.32 (1.10 to 1.58)], global *P* = 0.002. Among heterosexual men and women (*N* = 5330 observations), the effect of randomized arm on CLS-D differed according to time point (*P* = 0.017 for interaction). In a model including the interaction term, the effect of immediate versus deferred arm on CLS-D became greater with time: aOR (95% CI): 0.91 (0.66 to 1.26), 1.36 (1.00 to 1.85) and 1.66 (1.13 to 2.43) for months 4, 12 and 24, respectively. CLS-D did not differ with calendar year [aOR per later year: 1.05 (0.92 to 1.18) *P* = 0.48] but was higher for women compared with men [aOR (95% CI): 1.49 (1.15 to 1.92) *P* = 0.004].

Similar patterns over time were apparent when CLS was considered (Table 3 and Fig. 1b). Prevalence of CLS-D-HIV-risk decreased over time from month 4, as participants started ART (Table 3 and Fig. 1c). As expected, this decrease was particularly dramatic in the immediate ART group, resulting in substantial differences in CLS-D-HIV risk between randomized groups at months 12 and 24.

### Transmission risk beliefs

At month 12, participants in the immediate arm were more likely than those in the deferred arm to indicate a belief that a person on HIV treatment with undetectable viral load cannot, or is much less likely, to transmit HIV when having unprotected sex: 48.1 versus 40.4% for MSM [difference: 7.7%, 95% CI (−3.7 to 11.8%), *P* < 0.001, chi-squared test] and 36.7 versus 32.2% for heterosexuals [difference: 4.5%, 95% CI (0.03 to 8.9%), *P* = 0.049] (Table 3). Similar differences between arms were apparent at month 24 (Table 3).

For both MSM and heterosexuals, the proportion of participants who indicated this belief in a strong protective effect of viral suppression tended to increase over time from baseline in both arms. However, evidence that this belief was more likely with later calendar time was relatively weak. There was no trend in proportions reporting this belief over calendar time of questionnaire completion among MSM or heterosexuals in the immediate arm at month 12 or 24. In the deferred arm, there was some evidence of a trend at month 24: percentages of MSM reporting this belief were 43.8, 44.1, 47.4%, 57.1% for month 24 completion years 2011–2012, 2013, 2014 and 2015 respectively (*P* = 0.019 chi-squared test for trend). The corresponding percentages for heterosexuals were 26.8, 39.8, 43.9 and 36.6% (*P* = 0.058 for trend).

Transmission risk beliefs varied across regions. MSM recruited from high-income settings were more likely than those from low/middle income settings to indicate belief in a strong protective effect of viral suppression [53.6 versus 30.7% at month 12, *P* < 0.001; chi-squared test 58.3 versus 36.2% at month 24, *P* < 0.001]. However, the opposite was true for heterosexuals at month 12 [30.1% for high income versus 35.8% for low/middle income at month 12, *P* = 0.029] with no difference at month 24 [41.7 versus 41.1%, *P* = 0.84].

### Risk behaviour related to injection drug use

At baseline, 1.9% of MSM (40/2147) and 0.8% (15/1810) of heterosexual participants reported injecting recreational drugs in the past 2 months, of whom only three and four individuals, respectively reported injection drug use transmission risk. At month 12, 2% of MSM (48/2355) reported injection drug use: 1.6% in the immediate arm and 2.5% in the deferred arm, of whom two and one, respectively reported injection drug use transmission risk. For heterosexual individuals, 0.4% (7/1846) reported injection drug use at month 12, 0.7 and 0.1% in the immediate and deferred arms respectively, of whom one and zero, respectively reported injection drug use transmission risk.

## Discussion

Among MSM, compared with ART deferral until a CD4^+^ less than 350 cells/μl or AIDS, a strategy of immediate ART had no impact on prevalence of HIV-serodifferent CLS (CLS-D) over the subsequent 2 years, or on related measures of frequency of such sex or partner numbers. Among heterosexual men and women, the immediate ART strategy resulted in modestly higher prevalence and frequency of HIV-serodifferent CLS at 12 and 24 months compared with deferred ART. Among MSM and heterosexuals, the immediate ART strategy resulted in a substantial reduction in prevalence of HIV-transmission risk sex by month 12, as the vast majority of participants in this arm had viral suppression on ART and were therefore classified as no risk for HIV transmission, regardless of sexual behaviour.

CLS-D was the predefined primary endpoint in this study; this endpoint remains important for understanding the impact of early ART on condom use with serodifferent partners. However, the two additional endpoints are the most relevant for HIV and STI transmission. CLS-D-HIV-risk best captures HIV transmission-risk sex by accounting for viral suppression on ART, now known to be a critical factor in preventing transmission. CLS best captures risk of transmission or acquisition of other STIs, for which HIV-serostatus of partners, ART and HIV viral load are not relevant. These results illustrate the profound impact of early ART on HIV-transmission risk sex, which was less than 1% in the immediate ART arm by month 12 (compared with 11 and 8% for MSM and heterosexuals, respectively in the deferred ART arm) and was continued at this low level to month 24. Even though the immediate ART strategy resulted in a small increase in CLS-D among heterosexual participants at months 12 and 24, the impact of this was far outweighed by the high levels of viral suppression in this arm, which protected against HIV-transmission regardless of CLS-D. Even a very substantial increase in CLS-D would not have overturned the benefit conferred by early ART. Of note, this analysis suggested a benefit only from month 12, because the definition of CLS-D-HIV-risk required ART to have been started at least 6 months previously to confer protection from transmission. This was based on data from the Partners Prep study, which indicated a reduced but residual transmission risk persisting during the first 6 months of ART, because of incomplete viral suppression in blood and genital compartments [23]. In practice, and with newer ART drugs, viral suppression and subsequent protection may be attained at an earlier stage after ART initiation. But also of note, the difference between arms in CLS-D-HIV-risk attenuated from month 12 to month 24, and this would continue to occur as more individuals in the deferral arm started ART. In terms of STI transmission risk, the strategy of early ART resulted in a modest increase in prevalence of CLS among heterosexual individuals at year 2 only. However, less than 2% of heterosexual participants reported more than one CLS partner in the recall period. Risk of STI acquisition and transmission may be less concerning in this context. Incidence of bacterial STIs was not assessed in START.

Two previous randomized trials have assessed the impact of ART on sexual transmision risk [47,48]; neither supported the hypothesis that ART use leads to increased CLS. In the SMART trial (2002–2006), the prevalence of ‘high-risk behaviour’ (CLS-D or injecting risk behaviour) was similar compared between the continuous ART and CD4^+^-guided episodic ART arms [47]. Among the subgroup who was not on ART at baseline, there was a reduction in high-risk behaviour in the first 2 years in the continuous compared with episodic ART arm. More recently, in the TEMPRANO-ANRS12136 trial of immediate versus deferred ART initiation (2008–2012), the proportion of participants reporting CLS-D was similar when compared between randomized arms at year 1 [48]. Many observational studies have assessed the association between ART and sexual behaviour among people with diagnosed HIV [24–46], including two meta-analyses [29,32] and some studies in low/middle income countries [41,43,45]. The vast majority found no association of ART use or viral suppression with CLS-D, or found ART was in fact associated with lower levels of CLS-D. A few studies reported different findings overall, or in specific subgroups or analyses. Among sexually active women in the US Women's Interagency HIV Study (1996–2001), consistent condom use was less likely to be reported after ART initiation compared with pre-ART, in some adjusted models [24]. A small Australian study of HIV-serodifferent MSM couples (2001–2003) found higher levels of anal CLS-D among couples for whom the positive partner had suppressed viral load [25]. The Swiss HIV Cohort Study (2007–2009) found evidence of higher levels of CLS-D with stable partners among participants who were virally suppressed compared with those not on ART [26]. In a French study (2000–2009), in more recent years, ART use and suppressed viral load were associated with CLS-D among HIV-diagnosed heterosexual men with steady partners [27]. In the UK ASTRA study (2011–12), prevalence of CLS-D among MSM on ART was higher for those with self-reported undetectable viral load compared with those without [28]. Finally, a subsidiary analysis in one of the aforementioned meta-analyses found that an undetectable viral load was associated with slightly higher sexual risk-taking [29].

Therefore, previous literature suggested that, in some contexts, condom use may be influenced by knowledge of viral suppression, but the effect may be modest, and evidence is relatively weak in light of all relevant studies. These current results from START are the most contemporary (2010–2015) but seem broadly consistent with this synopsis, showing that starting ART may have modestly reduced condom use among heterosexuals. Although the combined heterosexual group was the predefined population of analysis, the pattern of results was broadly similar when men and women were examined separately (data not shown). The results from START and the other randomized trials have advantage that confounding is minimized in the comparison of the groups randomized to start or defer ART. It should also be noted that, as in the TEMPRANO trial, this START analysis addresses a slightly different question to the observational studies, as it evaluates the impact of starting ART on sexual behaviour over 2 years among people with high CD4^+^ counts. In most of the observational comparisons, people on ART varied in terms of time since starting treatment and CD4^+^ count; conceivably impact on sexual activity and condom use may vary according to these factors. However, in START, there was no evidence that the intervention effect on CLS-D varied according to time since diagnosis in heterosexual individuals or MSM.

There may be a number of reasons why, in START, the effect of early ART on CLS-D was apparent only in the heterosexual group. Patterns of partnerships differed for heterosexuals compared with MSM, with the vast majority having only one CLS partner during the recall periods. Possibly, trial participants were more likely to be given advice about the protection from transmission conferred by viral suppression in the context of stable serodifferent relationships. Some advice may have been related to desire for conception, resulting in higher CLS-D among heterosexual participants on ART, and may in part explain why the intervention effect on CLS-D was greater among those aged under 40 years. Furthermore, the month-12 time point was during or after 2011 but before 2014 for the majority of participants, giving potential for awareness of results from HPTN 052 related to heterosexual transmission, but not for substantive evidence relating to MSM (results from PARNER, Opposites Attract and PARNTER2 first being presented in 2014–2018). However, even at year 2 of follow-up, only about half of MSM and less than half of heterosexuals expressed belief in any substantial level of protection conferred by viral suppression; this is consistent with findings from other quantitative [28] and qualitative [21] studies. Although, in START, there was some evidence of increasing proportions expressing this belief over calendar time, trends were not marked or consistent across trial arms. Advice and information given to participants regarding viral suppression and transmission risks may also have differed across countries and clinical sites. Indeed, there was evidence of significant variation in transmission risk beliefs by recruitment setting, which differed for MSM and heterosexuals. MSM from high-income countries were more likely than those from low-income/middle-income countries to express belief in a strong protective effect of viral suppression, whereas for heterosexuals, there was some evidence of the opposite effect (belief more prevalent in low-income/middle-income settings). The trial was carried out over a period of considerable change with regard to scientific evidence available on this issue, and publicity surrounding it, and these complex trends are likely to reflect this. Increasing emphasis on the ‘U = U’ message [17] may be leading to further changes in transmission risk beliefs and patterns of condom use; it is likely that the full effect of the current research evidence is yet to be apparent.

Among MSM, in both the immediate and deferred treatment arms, CLS-D prevalence fell from randomization to months 4 and 12, and subsequently increased back towards the revised baseline levels by year 2. Among heterosexual participants, a similar but less marked decline in CLS-D occurred from revised baseline to month 4. Other studies have reported on temporary reductions in sexual risk for MSM following HIV diagnosis [51,52], and so a similar pattern may have occurred for those individuals in START who were newly diagnosed at trial entry. In addition, for all participants, trial participation and regular contact with healthcare professionals and services, may have had a moderating effect on levels of CLS.

Limitations of this study include the possibility of error or bias arising from self-reported sexual behaviour and incomplete or missing questionnaires, though 12-month response rates were high and similar between randomised arms, and questionnaires were self-completed. Participation in a trial with repeated monitoring of behavioural outcomes may have influenced condom use or its reporting. The measure of HIV-transmission risk sex assumes that the latest viral load is applicable to the entire 2-month recall period.

In conclusion, a strategy of early ART did not impact on levels of serodifferent CLS among MSM and resulted in a small increase among heterosexual individuals. However, in both groups, the strategy had a substantial favourable impact on HIV transmission risk behaviour at 1 year and is therefore, likely to result in a marked reduction in new HIV infections in the initial period. The modest increase in CLS among the heterosexual group suggests the importance of continuing to monitor sexual behaviour as ART use expands, in order to understand any impact on other sexually transmitted infections. Patterns of CLS may have changed with increasing promotion of the U = U message since the START trial was conducted.

## Acknowledgements

We would like to thank the START participants without whom this work would not be possible.

For a complete list of START investigators see *N Engl J Med* 2015; 373:795–807.

The START study is registered at clinicaltrials.gov (NCT00867048).

Author contributions: F.C.L.; A.J.R.; W.B.; A.G.; G.F.; W.E.S.; J.N.; S.E.; A.P. contributed to questionnaire design and conceptualization of the analysis. F.L. performed the analysis and drafted the manuscript. All authors contributed to data interpretation and revision of the manuscript. G.M.O.; J.M.M.; C.O.; J.Ga.; J.Ge.; K.R.; M.B.d.S. were clinical site principle investigators for the STRAT trial; S.E. was the regional co-ordinator; J.N. was the INSIGHT Primary Investigator.

Funding: The START study was primarily funded by the National Institute of Allergy and Infectious Diseases of the National Institutes of Health under Award numbers: UM1-AI068641 and UM1-AI120197. START was supported by the National Institutes of Health Clinical Center, National Cancer Institute, National Heart, Lung, and Blood Institute, Eunice Kennedy Shriver National Institute of Child Health and Human Development, National Institute of Mental Health, National Institute of Neurological Disorders and Stroke, National Institute of Arthritis and Musculoskeletal and Skin Diseases, Agence Nationale de Recherches sur le SIDA et les Hépatites Virales (France), National Health and Medical Research Council (Australia), National Research Foundation (Denmark), Bundes ministerium für Bildung und Forschung (Germany), European AIDS Treatment Network, Medical Research Council (United Kingdom), National Institute for Health Research, National Health Service (United Kingdom), and University of Minnesota. Antiretroviral drugs were donated to the central drug repository by AbbVie, Bristol-Myers Squibb, Gilead Sciences, GlaxoSmithKline/ViiV Healthcare, Janssen Scientific Affairs and Merck.

### Conflicts of interest

G.M.C. has done paid consultancy work for Gilead Sciences, ViiV Healthcare and Mylan. J.M.M has received grants from Gilead and honoraria for advisory boards with Gilead, Merck and ViiV. J. Ga. has received honoraria for lectures and advisory boards, and has institution (Hospital Clinic de Barcelona) research grants from ViiV, Gilead, Janssen and MSD. Since May 2018 J Ga is a full time employee of ViiV Healthcare. F.C.L., A.J. R., W.B., A.G., G.F., W.E.S.,J.N., S.E.,C.O., J. Ge., K.R., A.N.P have no conflicts of interest.
